# Cell proliferation and invasion are regulated differently by EGFR and MRP1 in T-DM1-resistant breast cancer cells

**DOI:** 10.1038/s41598-019-52797-z

**Published:** 2019-11-08

**Authors:** Yukinori Endo, Sarah Lyon, Yi Shen, Nishant Mohan, Wen Jin Wu

**Affiliations:** Division of Biotechnology Review and Research I, Office of Biotechnology Products, Office of Pharmaceutical Quality, Center for Drug Evaluation and Research, U.S. Food and Drug Administration (FDA), Silver Spring, MD 20993 USA

**Keywords:** Cancer therapeutic resistance, Target identification

## Abstract

We recently reported that T-DM1-resistant JIMT1 (T-DM1R-JIMT1) cells exhibited high invasive activity via EGFR and integrin cooperated pathways and gained cross-resistance to doxorubicin. Here, we show that EGFR positively coordinates with MRP1 in T-DM1R-JIMT1 cells to contribute to cross-resistance to doxorubicin. Downregulating EGFR and MRP1 inhibits T-DM1R-JIMT1 cell growth and re-sensitizes T-DM1R cells to doxorubicin, suggesting that dual targeting EGFR and MRP1 could serve as a therapeutic approach to overcome T-DM1 resistance. However, it increases cell invasion activity of T-DM1R-JIMT1 cells with molecular and cellular phenotypes similar to the breast cancer cells that express low levels of HER2 (MDA-MB-231 and BT-549 cells). Importantly, the invasion activity of MDA-MB-231 and BT-549 cells is also significantly increased after chronically exposed to T-DM1 although cell growth of MDA-MB-231 and BT-549 cells is not inhibited by T-DM1. These results highlight the importance of HER2 heterogenicity in HER-positive breast cancers treated with T-DM1. Our study also provides evidence demonstrating that proliferation and invasion activities of T-DM1R-JIMT1, and MDA-MB-231 and BT-549 cells are regulated by different mechanisms and that different aspects of cancer cell behaviors affected by targeted-therapeutics should be fully characterized in order to overcome T-DM1-resistant disease and to prevent cancer metastasis.

## Introduction

Ado-trastuzumab emtansine (also known as T-DM1) is an antibody-drug conjugate (ADC) for patients with HER2-positive metastatic breast cancer whose disease has progressed on trastuzumab plus chemotherapy^1^. T-DM1 consists of trastuzumab, a humanized monoclonal antibody targeting HER2, and DM1, a maytansinoid-derived cytotoxic agent, that are conjugated via non-reducible thioether linker^2^. The mechanism of action associated with the ADC is that T-DM1 targets HER2 overexpressed on the cell surface of breast cancers via trastuzumab, and subsequently T-DM1/HER2 complexes are internalized into lysosomes where antibody component of T-DM1 is degraded followed by the release of Lys-MCC-DM1 into the cytoplasm^3,4^. Lys-MCC-DM1 then targets microtubules and blocks microtubular polymerization, resulted in apoptosis of cancer cells^3,5–7^.

Despite initial favorable outcomes, most patients treated with T-DM1 eventually develop T-DM1-resistant diseases^8^. Pre-clinical studies demonstrate that the T-DM1-resistant breast cancer cells appear cross-resistant to standard-of-care (SOC) chemotherapeutics^9–11^, which is accompanied by the enhanced metastatic potential^10^. Pre-clinical studies have also revealed multiple mechanisms, including a decrease in HER2 overexpression in HER2-positive breast cancer cells, contribute to resistance to T-DM1^9–12^, while no major changes in HER2 expression in T-DM1-resistant clones, which are derived from HER2-positive breast cancer cells (BT-474), are observed compared with BT-474 parental cells^12^. Li *et al*. (2018) and our group found that epidermal growth factor receptor (EGFR) was upregulated in T-DM1-resistant breast cancer cells^10,11^. However, it remains largely unknown as to how T-DM1-resistant breast cancer cells exhibit the enhanced metastatic potential.

Integrins are well-known cell surface receptors for extracellular matrix (ECM) proteins and contribute to cancer progression and invasion^13,14^. Integrins are also known to share common signaling networks with receptor tyrosine kinases (RKTs) such as EGFR and play critical roles in therapeutic resistance to therapies targeting RTKs and their downstream signaling molecules in cancer^15^. We previously demonstrate that α5β1 integrins are upregulated by EGFR and that α5β1 integrin blockage enhances cell invasion activity in T-DM1-resistant cells due to increase in αVβ3 integrin activity^10^. Thus, we proposed a dual targeting of EGFR and integrins for the treatment of T-DM1-resistant disease^10^.

ATP-binding cassette (ABC) transporter family members play an important role in multiple drug resistance (MDR)^16–18^. Since the ABC transporters such as MDR1 and multidrug resistance-associated protein 1 (MRP1) appear upregulated in T-DM1-resistant breast cancer cells^9–11^, it is possible that these ABC transporters are involved in both acquired resistance to T-DM1 and cross-resistance to SOC chemotherapeutics and regulate invasive behavior of T-DM1-resistant breast cancer cells. Delineating the complicated relationships among EGFR, MRP1 and α5β1 integrins in T-DM1-resistant breast cancer cells may lead to a better understanding of biological consequences resulting from the dysregulation of these critical molecules and development of novel combination therapies to prevent or overcome T-DM1-resistant disease.

## Results and Discussion

**Figure 1:**
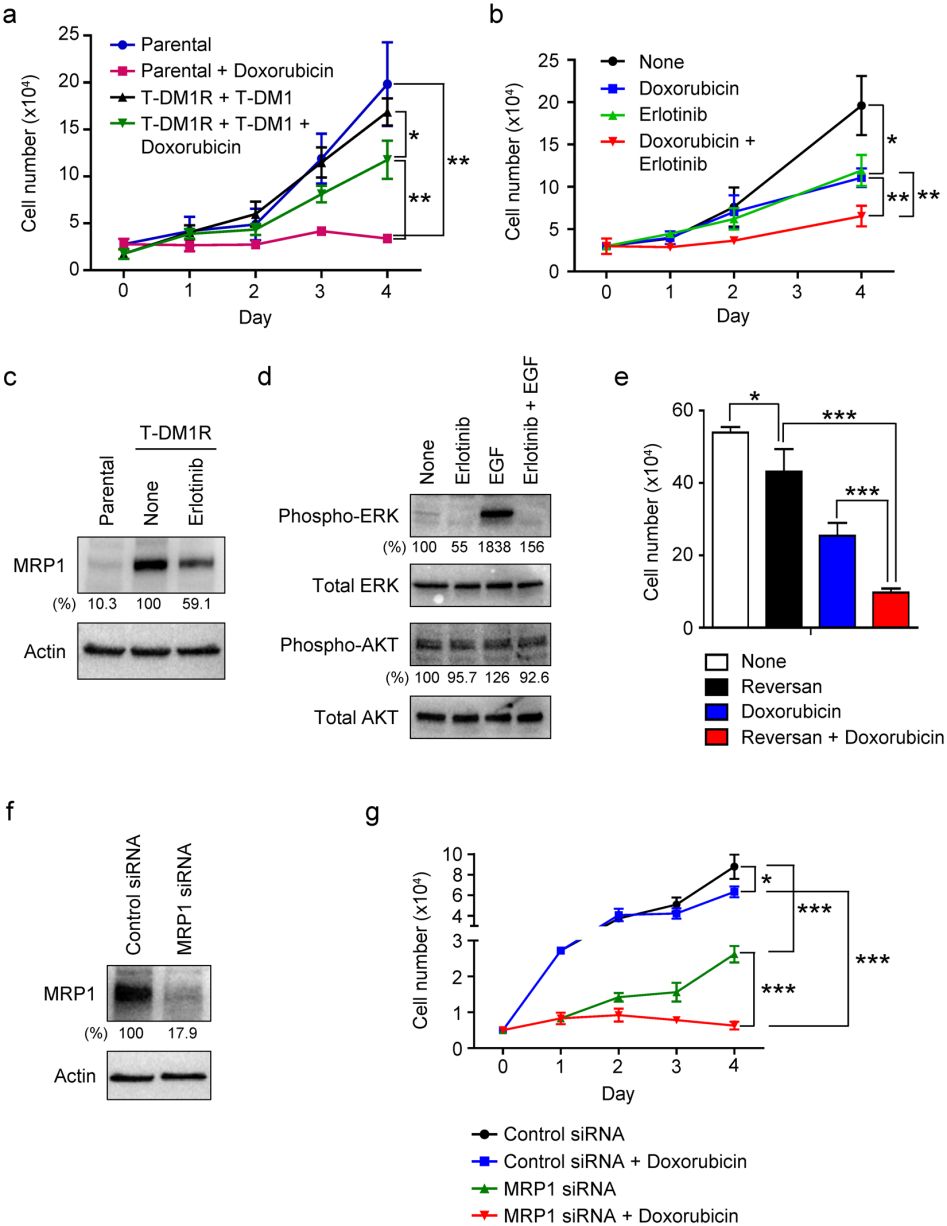
MRP1 is upregulated by EGFR activity and involved in cross-resistance to doxorubicin in T-DM1R-JIMT1 cells. (**a**) Cell growth profiles of JIMT parental and T-DM1R-JIMT1 cells treated with 50 nM Dox. T-DM1R-JIMT1 cells were cultured in the presence of 4 µg/ml of T-DM1. Parental vs. Parental + Dox: *p*-value, 0.0021; T-DM1R-JIMT1 + T-DM1 vs. T-DM1R + T-DM1 + Dox: *p*-value, 0.0120; Parental + Dox vs. T-DM1R + T-DM1 + Dox: *p*-value, 0.0014. (**b**) Cell growth profiles of T-DM1R-JIMT1 cells treated with either 5 µM erlotinib or 50 nM doxorubicin, or both drugs. T-DM1R-JIMT1 cells were cultured in the media containing 4 µg/ml of T-DM1. None vs. erlotinib: *p*-value, 0.0119; Dox vs. Dox + erlotinib: *p*-value, 0.0044; erlotinib vs. Dox + erlotinib: *p*-value, 0.0066. (**c**) The levels of MRP1 were evaluated by Western blot in the WCL of JIMT1 parental and T-DM1R-JIMT1 cells treated with either 5 µM erlotinib or left untreated. The quantitative levels of MRP1 detected by Western blot analysis were done by ImageJ (NIH). From here on, all quantitation of Western blot analysis was performed using ImageJ (NIH). (**d**) The levels of phosphorylated ERK and AKT, and total ERK and AKT in WCL of T-DM1R-JIMT1cells were evaluated by Western Blot analysis. Overnight serum-starved T-DM1R cells were preincubated with 5 µM erlotinib for 1 hour prior to 100 ng/ml EGF stimulation for 45 minutes. From here on, actin or GAPDH blots were used as a control for the densitometric quantification, and the numbers for the bands were normalized by actin or GAPDH. (**e**) Cell growth of T-DM1R-JIMT1 cells treated with either 2.5 µM reversan or 50 nM doxorubicin, or both drugs at Day 4 time point. T-DM1R-JIMT1cells were cultured in the media containing 4 µg/ml of T-DM1. None vs. Reversan: *p*-value, 0.0445; Reversan vs. Reversan + Dox: *p*-value, 0.0004; Dox vs. Reversan + Dox: *p*-value, 0.0009. (**f**) Knock-down efficiency of MRP1 in T-DM1R-JIMT1 cells was evaluated by Western blot analysis. (**g**) Cell growth profiles of T-DM1R-JIMT1 cells treated with control siRNA or MRP1 siRNA, +/− 50 nM Dox. Control siRNA vs. Control siRNA + Dox: *p*-value, 0.0154; Control siRNA vs. MRP1 siRNA: *p*-value, 0.0005, MRP1 siRNA vs. MRP1 siRNA + Dox: *p*-value, <0.0001; Control siRNA + Dox vs. MRP1 siRNA + Dox: *p*-value, <0.0001.

Using JIMT1 cells, which have been commonly used as a cellular model to study the mechanisms of T-DM1 resistance^9,10^, we previously showed that T-DM1-resistant JIMT1 (designated as T-DM1R-JIMT1) cells acquired cross-resistance to chemotherapeutic drugs such as paclitaxel and doxorubicin (Dox)^10^. Figure 1a provided an additional example showing that T-DM1R-JIMT1cells exhibited resistance to Dox as compared to that of parental cells. We then examined whether EGFR activity was involved in the cross-resistance to chemotherapeutic drugs. As shown in Fig. 1b, after T-DM1R cells were treated with both Dox and erlotinib (a tyrosine kinase inhibitor for EGFR), cell growth was significantly inhibited as compared with that of T-DM1R-JIMT1 cells treated with either Dox or erlotinib. These results indicate that the increased EGFR activity is required for acquiring cross-resistance to Dox in T-DM1R-JIMT1 cells.

MRP1 and MDR1 are members of ABC transporters and believed to be involved in multiple drug resistance (MDR)^16–18^. It has been shown that EGF-induced activation of EGFR upregulates ABC transporters in a breast cancer cell line, MCF-7^19^. However, the functional correlation between EGFR and ABC transporters to modulate cell invasion activity has not been examined in T-DM1R cells. We previously showed that MRP1 expression was dramatically increased in T-DM1R-JIMT1 cells, whereas MDR1 expression was not detected in both parental and T-DM1R-JIMT1 cells^10^. After 48 hrs treatment with erlotinib, MRP1 protein expression was decreased in T-DM1R-JIMT1 cells compared with non-treated cells (Figs. 1c, S1c. Note: From here on, all unprocessed original Western blot images used in the figures of this article are available in Supplemental information). Figure 1d provided evidence that erlotinib inhibited the downstream of EGFR signaling such as phosphorylation of ERK. Interestingly, AKT appeared not responding EGF stimulation in T-DM1R-JIMT1 cells. Nevertheless, these results suggest that the activity of EGFR is involved in upregulating MRP1 expression in T-DM1R-JIMT1 cells. We then addressed if MRP1 was involved in cross-resistance to chemotherapeutic drug in T-DM1R-JIMT1 cells. Reversan is the MRP1 specific inhibitor that blocks MRP1 activity without changing its expression^20^. As shown in Fig. 1e, the growth of T-DM1R-JIMT1cells was reduced when cells were treated with the reversan as compared with that of cells left untreated. Moreover, the inhibitory effect on cell growth was even more significant when cells were treated with both Dox and reversan as compared with either Dox or reversan alone, indicating that inhibition of MRP1 increases the sensitivity of T-DM1R-JIMT1 cell to Dox. These results were further confirmed by an alternative approach, i.e., RNAi. Figure 1f showed that MRP1 was 82.1% knocked down by specific siRNA in T-DM1R-JIMT1 cells. The effect of silencing MRP1 on cell sensitivity to Dox was then examined. As shown in Fig. 1g, the growth rate of MRP1-silenced T-DM1R cells was reduced as compared to that of control siRNA-treated T-DM1R cells. This result is consistent with data shown in Fig. 1e, suggesting that MRP1 plays a role in the regulation of T-DM1R-JIMT1 cell growth. Silencing MRP1 dramatically increased the sensitivity of the T-DM1R cells to Dox such that no cell growth was observed for MRP1-silenced T-DM1R-JIMT1 cells treated with Dox, whereas the control siRNA-treated T-DM1R-JIMT1 cells exhibited cross-resistance to Dox (Fig. 1g). Taken together, these data suggest that blockage of MRP1 re-sensitizes T-DM1R-JIMT1 cells to Dox and that combination of reversan and Dox may be a novel therapeutic approach to overcome T-DM1 resistance.

**Figure 2:**
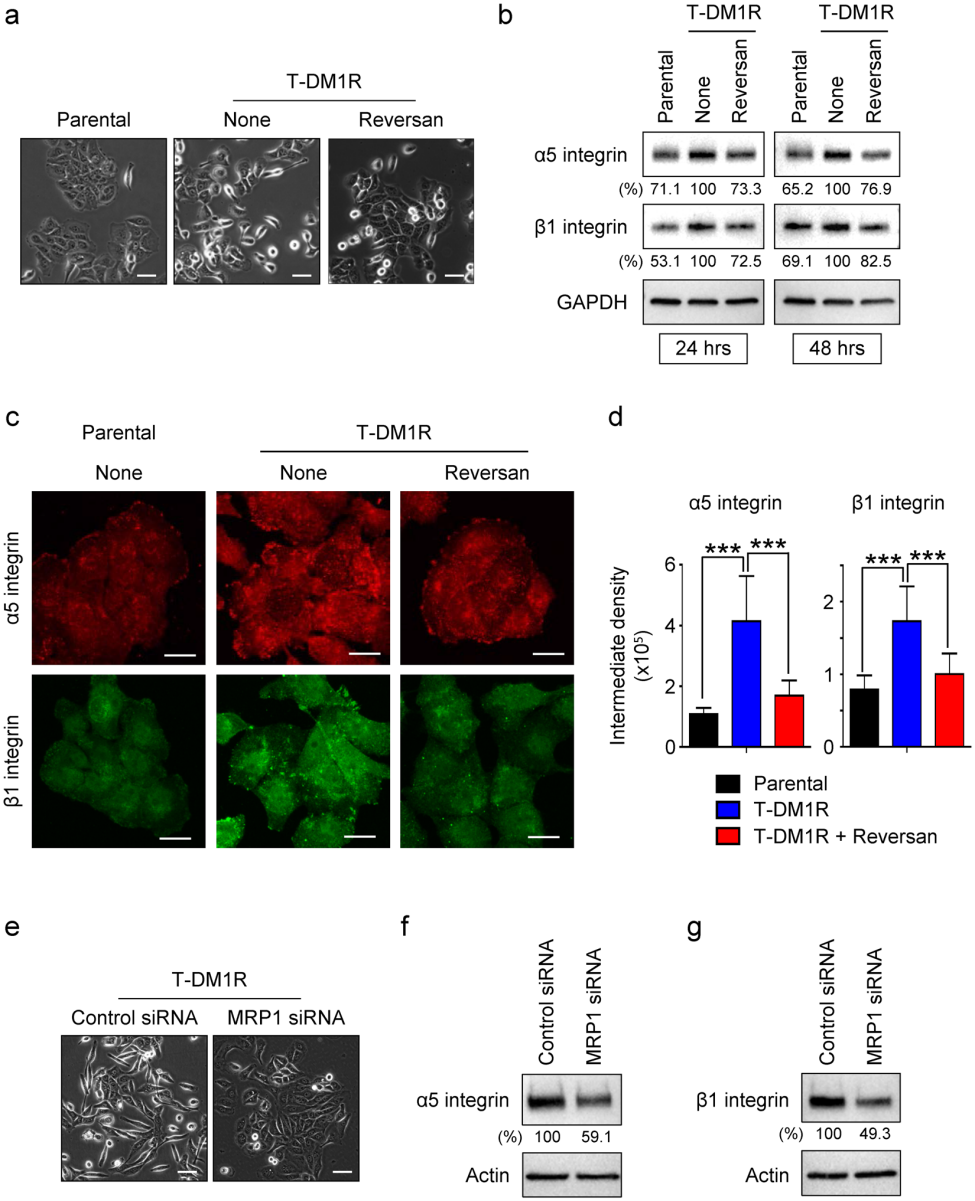
MRP1 upregulates α5β1 integrin in T-DM1R-JIMT1 cells. (**a**) Bright field (BF) images of JIMT1 parental and T-DM1R-JIMT1 cells treated with either 2.5 µM reversan for 48 hrs or left untreated. Scale bar, 50 µm. (**b**) 1 × 10^6^ JIMT1 parental or T-DM1R-JIMT1 cells were seeded on fibronectin-coated 6-well plates and cultured overnight. The levels of α5 and β1 integrin expressions in the WCL collected 24 and 48-hour time points were evaluated by Western blot analysis. (**c**) Fluorescent immunostaining images showing β1 integrin and α5 integrin in JIMT1 parental and T-DM1R-JIMT1 cells treated with either reversan for 48 hrs or left untreated. Scale bar, 20 µm. (**d**) Quantification of intermediate density for fluorescent immunostaining images in Fig. 2c by ImageJ (NIH). For α5 integrin, parental vs. T-DM1R: *p*-value, <0.0001; T-DM1R vs. T-DM1R + Reversan: *p*-value, <0.0001. For β1 integrin, parental vs. T-DM1R: *p*-value, <0.0001; T-DM1R vs. T-DM1R + Reversan: *p*-value, 0.0001. (**e**) BF images of control and MRP1 siRNA-treated T-DM1R-JIMT1 cells after 48 hrs post siRNA transfection. Scale bar, 50 µm. (**f**) The levels of α5 integrin expression were examined in WCL of control and MRP1 siRNA-treated T-DM1R-JIMT1 cells by Western blot analysis. (**G**) The levels of β1 integrin expression were examined in WCL of control and MRP1 siRNA-treated T-DM1R-JIMT1 cells by Western blot analysis.

Integrins are predominant and well-characterized cell surface receptors for extracellular matrix proteins such as fibronectin, laminin and collagen, which control cell motility and cell invasive activity^13,21,22^. We previously found that the increased EGFR activity upregulated α5β1 integrin expression that promoted cell motility in T-DM1R-JIMT1 cells^10^. We also reported that silencing β1 integrin by siRNA in T-DM1R-JIMT1 cells destabilized α5, but increased expression of αV, a critical integrin mediating the invasion and metastases in many different cancers, resulting in an increased invasion activity in T-DM1R-JIMT1 cells^10^. We next investigated the functional connection between MRP1 and α5β1 integrin. After 24 hrs treatment with reversan, the morphology of T-DM1R-JIMT1 cells was changed from the mesenchymal-like spindle-shape to an epithelial-like tile-shape similar to that of parental cells (Fig. 2a). These morphological changes were previously also observed when β1 integrin expression was silenced by siRNA or EGFR activity was inhibited by erlotinib in T-DM1R-JIMT1 cells^10^. Thus, data shown in Fig. 2a suggest that MRP1 might regulate α5β1 integrin function in T-DM1R-JIMT1 cells. Figure 2b showed that the increase in α5β1 integrin expression in T-DM1R-JIMT1 cells, compared to those in parental cells, was decreased by reversan on fibronectin-coated dishes. The decrease in α5β1 integrin protein expression was also confirmed by fluorescent immunostaining (Fig. 2c,d). These results were further confirmed by siRNA technology. When MRP1 expression was knocked down by siRNA, cell morphology was also changed from the mesenchymal-like spindle-shape to an epithelial-like tile-shape similar to reversan-treated cell morphology (Fig. 2e). Furthermore, both α5 and β1 integrin protein expression were decreased in the MRP1-knocked down T-DM1R-JIMT1 cells compared with those in control siRNA-treated T-DM1R-JIMT1 cells (Fig. 2f,g). These results provide strong evidence demonstrating that increased MRP1 activity is required for upregulating these integrin expressions in T-DM1R cells and revealed a novel cellular function for MRP1 in the regulation of integrins.

**Figure 3:**
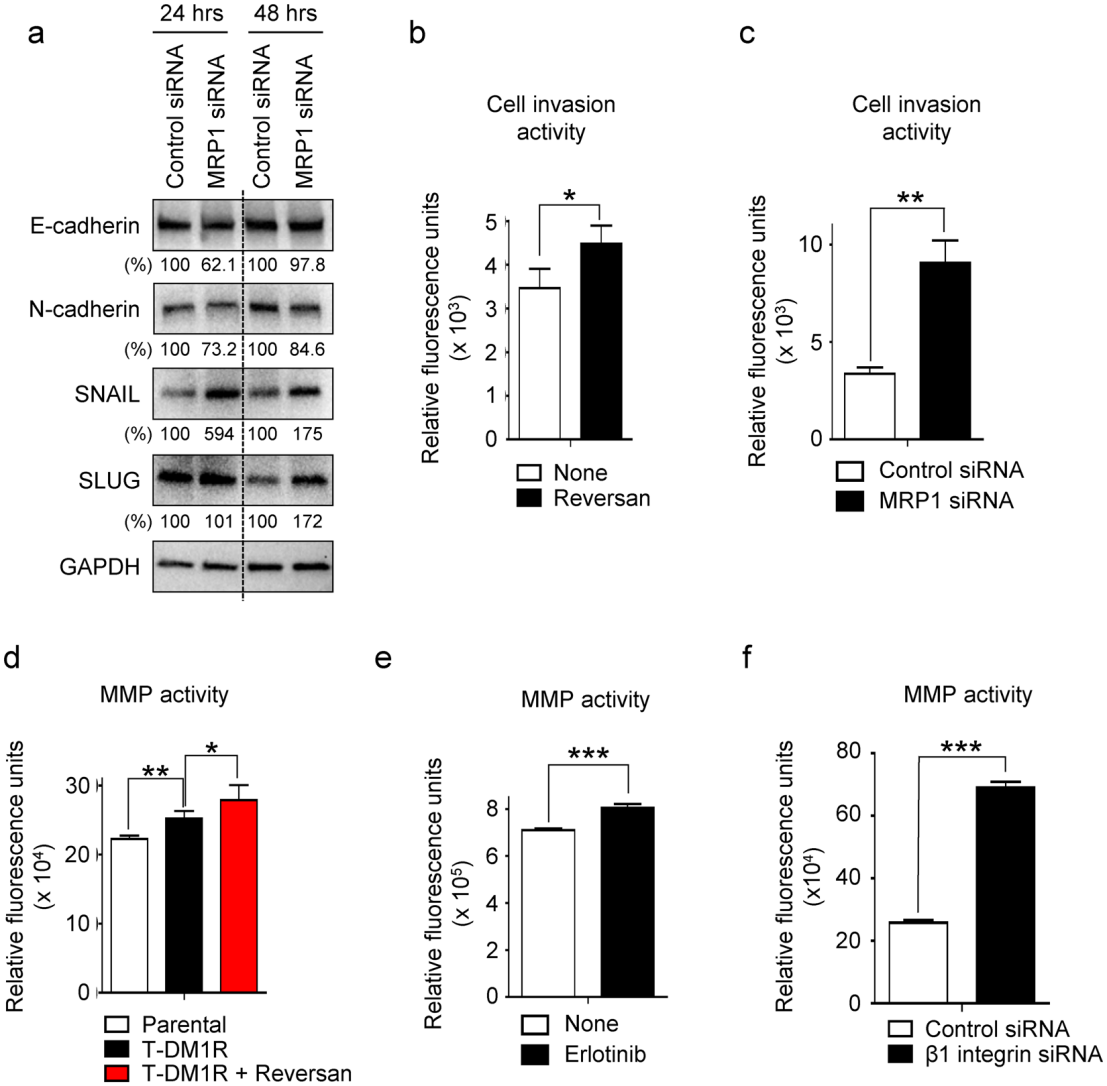
Blockage of MRP1 enhances MMP activity and cell invasion activity by down-regulating α5β1 integrin in T-DM1R-JIMT1 cells. (**a**) The levels of E-cadherin, N-cadherin, SNAIL, and SLUG were evaluated by Western blot in the WCL of T-DM1R-JIMT1 cells treated with either control siRNA or MRP1 siRNA. (**b**) Cell invasion activity of T-DM1R-JIMT1 cells treated with either 2.5 µM reversan for 48 hrs or left untreated. None vs. Reversan: *p*-value, 0.0078. (**c**) Cell invasion activity of T-DM1R-JIMT1 cells treated with either control siRNA or MRP1 siRNA. Control siRNA vs. MRP1 siRNA: *p*-value, 0.0006. (**d**) Total MMP activities were measured in the WCL of either JIMT1 parental, T-DM1R-JIMT1, or Reversan-treated T-DM1R-JIMT1 cells. Parental vs. T-DM1R: *p*-value, 0.0014; T-DM1R vs. T-DM1R + Reversan: *p*-value, 0.0337. (**e**) Total MMP activities were measured in the WCL of either none-treated or erlotinib-treated T-DM1R-JIMT1 cells. None vs. erlotinib: *p*-value, 0.0003. (**f**) Total MMP activities were measured in the WCL of either control siRNA-treated or β1 integrin siRNA-treated T-DM1R-JIMT1 cells. Control siRNA vs. β1 integrin siRNA: *p*-value, <0.0001.

We previously showed that when function of β1 integrin was inhibited by either siRNA or Mab 13, a monoclonal antibody that binds and inhibits α5β1 integrin function, the invasion activity of T-DM1R-JIMT1 cells was significantly increased^10^. This was highly likely mediated by increased αVβ3 integrin activity since this enhanced cell invasion activity can be blocked by either RGD peptide, an inhibitor for αVβ3 integrins or αV integrin siRNA^10^. In addition, we confirmed the involvement of αVβ3 integrin in the enhanced cell invasion by demonstrating that Cilengitide, a specific inhibitor for αVβ3 and αVβ5 integrins, reduced the increased cell invasion activity (data not shown) in T-DM1R-JIMT1 cells. Since cell morphological change was observed in MRP1-silenced T-DM1R-JIMT1 cells and MRP1 activity was positively correlated with α5β1 integrins (Fig. 2e–g), we examined epithelial-to mesenchymal transition (EMT) markers, including E-cadherin, N-cadherin, SNAIL and SLUG in MRP1-silenced T-DM1R-JIMT1 cells. As shown in Fig. 3a, SNAIL and SLUG expressions were increased in MRP1-silenced T-DM1R-JIMT1 cells while N-cadherin was decreased compared to T-DM1R-JIMT1 cells treated with control siRNA. These data supported the idea that inhibition of MRP1 enhanced cell invasion activity of T-DM1R-JIMT1 cells. As expected, inhibition of MRP1 activity by reversan significantly enhanced T-DM1R-JIMT1 cell invasion activity compared with the untreated cells (Fig. 3b). As an alternative approach, silencing MRP1 by MRP1 siRNA also significantly enhanced T-DM1R-JIMT1 cell invasion activity compared with control siRNA-treated T-DM1R-JIMT1 cells (Fig. 3c). Matrix metalloproteinase (MMP) activities play an important role in cancer metastatic process by proteolytic degradation of ECMs^23^. Thus, we investigated total MMP activities in reversan-treated T-DM1R cells. As shown in Fig. 3d, total MMP activities were significantly increased in T-DM1R-JIMT1 cells compared to that in parental cells. Furthermore, the treatment of reversan caused further enhancement of MMP activities in T-DM1R-JIMT1 cells (Fig. 3d), consistent with data shown in Fig. 3a–c. These data suggest that increased MMP activities result in the increased cell invasion. Figure 3e,f showed that total MMP activities were also significantly increased in either erlotinib-treated or β1 integrin-silenced T-DM1R-JIMT1 cells. Taken together, these results support the functional correlation between MRP1 and α5β1 integrins to mediate cell invasion in T-DM1R-JIMT1 cells.

**Figure 4:**
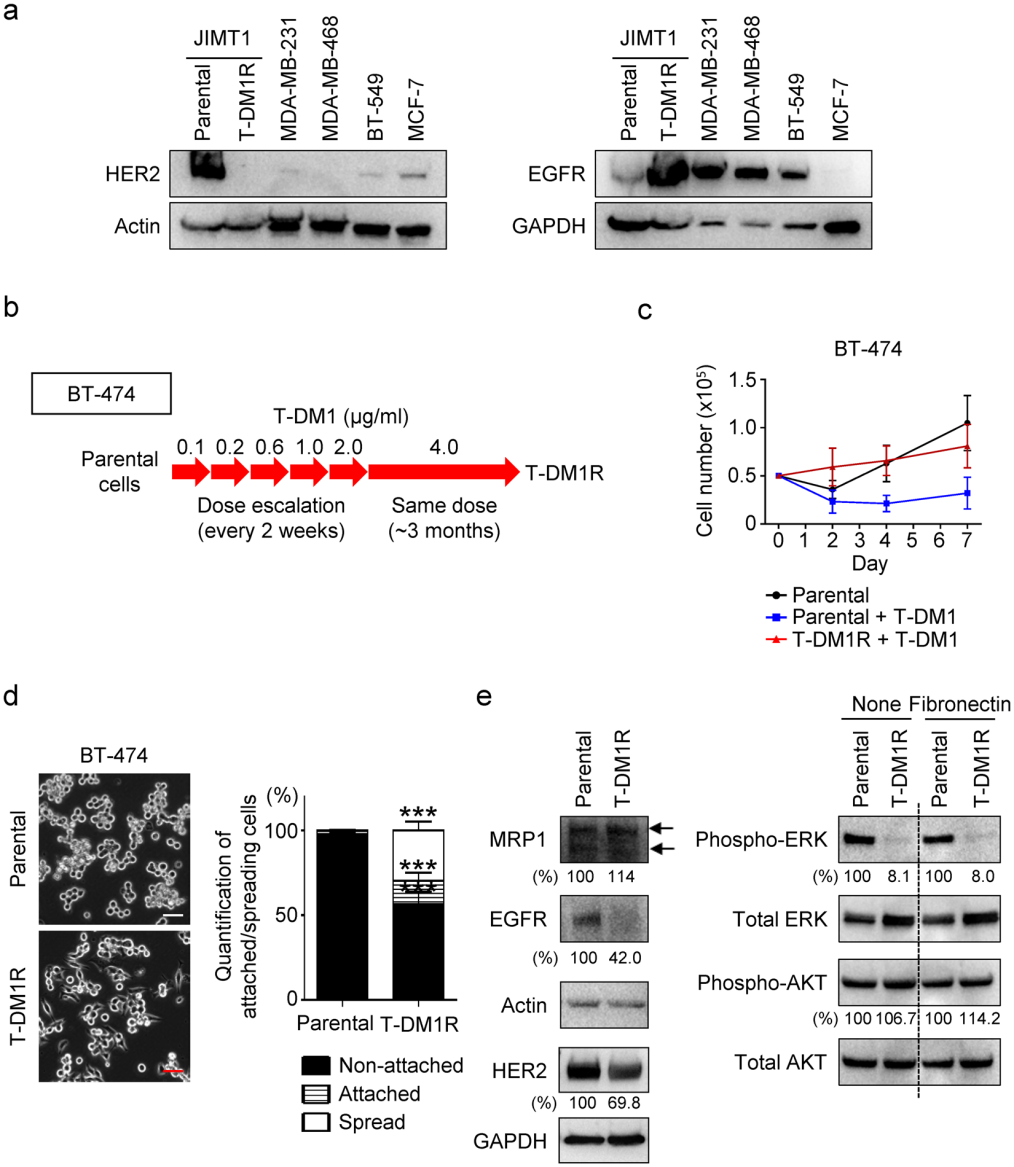
Establishment of T-DM1-resistant cells from BT-474 cell line. (**a**) The levels of HER2 and EGFR protein expression in the indicated cells were measured using Western blot analysis. (**b**) Procedures and the time lines for developing and establishing T-DM1-resistant (T-DM1R) cells from the indicated breast cancer cell line. T-DM1R cells were maintained in the presence of T-DM1 (4 µg/ml of T-DM1). (**c**) Cell growth profiles of parental and T-DM1R-BT-474 cells. Parental or T-DM1R-BT-474 cells were cultured in the media containing T-DM1 as indicated in (**b)**. (**d**) Images of attached/spread cells on fibronectin pre-coated wells after 4 hrs post seeding and quantification of ratio (%) of non-attached/attached/spread cells in parental BT-474 and T-DM1R-BT-474 cells. Scale bar, 50 µm. For parental BT-474 vs. T-DM1R-BT-474, no attachment: *p*-value, <0.0001; attached: *p*-value, 0.0002; spread: *p*-value, <0.0001. (**e**) The levels of MRP1, EGFR, and HER2 expressions, and downstream of EGFR signaling, i.e., phospho-ERK and phospho-AKT were evaluated by Western blot in the WCL of BT-474 parental and T-DM1R cells.

Next, we addressed the question of whether the functional correlation between MRP1 and α5β1 integrins is conserved in T-DM1R cells derived from HER2-positive/trastuzumab-sensitive breast cancer cells BT-474 (designed as T-DM1R-BT-474). Figure 4a showed levels of HER2 (left panels) and EGFR (right panels) expression in the indicated breast cancer cell lines. We previously showed that HER2 expression in BT-474 cells is about 3–4 folds higher than that in JIMT1 cells^24^. Figure 4b showed the procedures and time lines for establishing T-DM1-resistant BT-474 cells. Figure 4c showed that BT-474 cells became resistant to T-DM1 treatment after these cells were chronically exposed to T-DM1 for the time indicated in Fig. 4b. We further examined the cellular behavior differences between the parental and resistant cells using a cell spreading assay. As shown in Fig. 4d, T-DM1-resistant cells demonstrated faster cell spreading than that of parental cells on fibronectin-coated dish (Fig. 4d). The changes in HER2, EGFR and MRP1 expressions in T-DM1R-BT-474 cells were then examined. Figure 4e showed that the expression of MRP1 was modestly increased in T-DM1R-BT-474 cells as compared to that in parental cells. While HER2 expression in T-DM1R-BT-474 cells was about 30% reduction compared with that in parental cells, EGFR expression was also reduced (~ 60%) compared to that in parental cells. Additionally, ERK phosphorylation was reduced in T-DM1R-BT-474 cells, which may be the consequence of the reduced EGFR and HER2 expression in T-DM1R cells (Fig. 4e). This is different from that in T-DM1R-JIMT1 cells where EGFR expression and downstream of EGFR signaling such as phosphorylation of ERK are increased when JIMT1 cells become resistant to T-DM1^10^. AKT phosphorylation appeared not changed in T-DM1R cells as compared to that in parental cells (Fig. 4e). Taken together, T-DM1R-BT-474 and JIMT1 cells exhibited some commonality in changes in some critical signaling molecules and also displayed differences in molecular phenotype, suggesting that these two cell lines acquire T-DM1 resistance via distinct mechanisms although the parental cells of these two resistant-cell lines are sensitive to T-DM1. It should be noted that BT-474 cells are sensitive to trastuzumab, whereas JIMT1 cells are primarily resistant to trastuzumab. This difference could contribute to the distinct mechanisms for these two cell lines to acquire T-DM1 resistance differently.

**Figure 5:**
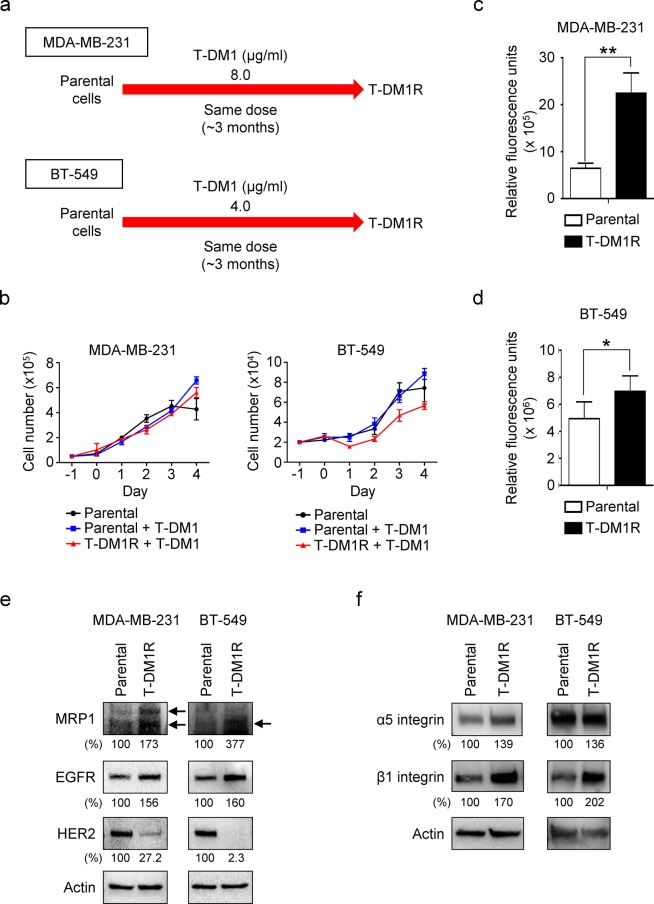
Cell invasion activity is significantly increased in T-DM1R-MDA-MB-231 and T-DM1R-BT-549 cells. (**a**) Procedures and the time lines for developing and establishing T-DM1-resistant (T-DM1R) MDA-MB-231 and BT-549 cells. T-DM1R-MDA-MB-231 and T-DM1R-BT-549 cells were maintained in the presence of T-DM1 (4 µg/ml of T-DM1 for T-DM1R-BT-549 cells, 8 µg/ml of T-DM1 for T-DM1R-MDA-MB-231 cells). (**b**) Cell growth profiles of MDA-MB-231 and BT-549 parental and their T-DM1R cells. Parental or T-DM1R cells were cultured in the media containing T-DM1 as indicated in Fig. 4b. (**c**) Cell invasion activity of parental cells and T-DM1R-MDA-MB-231 cells. Parental vs. T-DM1R-MDA-MB-231 cells: *p*-value, 0.0017. (**d**) Cell invasion activity of parental and T-DM1R-BT-549 cells. Parental vs. T-DM1R-BT-549 cells: *p*-value, 0.0224. (**e**) The levels of MRP1, EGFR, and HER2 were evaluated by Western blot in the WCL of MDA-MB-231 and BT-549 parental and T-DM1R-MDA-MB-231 and T-DM1R-BT-549 cells. (**f**) The levels of α5 and β1 integrins were evaluated by Western blot in the WCL of MDA-MB-231 and BT-549 parental and T-DM1R-MDA-MB-231 and T-DM1R-BT-549 cells.

Breast cancers are a heterogeneous disease among different patients (intertumor heterogeneity) and individual tumor (intratumor heterogeneity)^25^. Intratumoral HER2 heterogeneity was reported in 16–36% of HER2-positive breast cancer and associated with poor survival in HER2-positive breast cancer^26^. It was recently reported that HER2 heterogeneity is a predictor of response to T-DM1, implicating that HER2-targeted therapy, T-DM1 + pertuzumab, a HER2-trgeted monoclonal antibody, may be insufficient to completely eradicate a HER2-positive cancer^27^. Thus, this has raised a rationale to explore how breast cancer cells with relatively low HER2 expression, for example, MDA-MB-231 and BT-549 cells, respond to T-DM1 treatment. This study may lead to discovery of novel treatments for the HER2-positive breast cancer with HER2 heterogeneity. We then investigated the changes in MRP1, EGFR and HER2 expression in MDA-MB-231 and BT-549 cells that were chronically exposed to T-DM1 for the indicated time (designated as T-DM1R-MDA-MB-231 and T-DM1R-BT-549 cells) (Fig. 5a). The cell growth for parental and T-DM1-treated cells were not significantly inhibited by T-DM1 treatment (Fig. 5b). The cell invasion activity, however, was significantly increased in both T-DM1R-MDA-MB-231 and T-DM1R-BT-549 cells as compared with that in both parental cells (Fig. 5c,d). These results provide critical information regarding the treatment of HER2-positive breast cancers with HER2 heterogeneity and suggest that T-DM1 treatment is unable to inhibit the growth of cancer cells with low HER2 expression in HER-positive breast cancer. More importantly, T-DM1 treatment could potentially increase metastatic potential of the cells that express relatively lower HER2. The combination of T-DM1 with other therapy, e.g., chemotherapy, to inhibit low HER2 cancer cells in HER2-positive breast cancer may be under consideration to control cancer metastasis. Based on data obtained from MDA-MB-231 and BT-549 cells, we suggest that the concept of drug resistance is not only limited to resistance to growth inhibition induced by drug, but also the changes in other cellular behavior, e.g., cell invasion and other cancer metastatic potentials.

The changes in MRP1, EGFR and HER2 expression in T-DM1R-MDA-MB-231 and T-DM1R-BT-549 cells were largely similar to those in T-DM1R-JIMT1 cells (Fig. 5e). Specifically, the expressions of MRP1 and EGFR were increased and HER2 expression was reduced in the cells treated with T-DM1 (Fig. 5e left panels). We also found that α5 and β1 integrins were upregulated in T-DM1R-MDA-MB-231 and T-DM1R-BT-549 cells, which is consistent with that in T-DM1R-JIMT1 cells (Fig. 5f).

**Figure 6:**
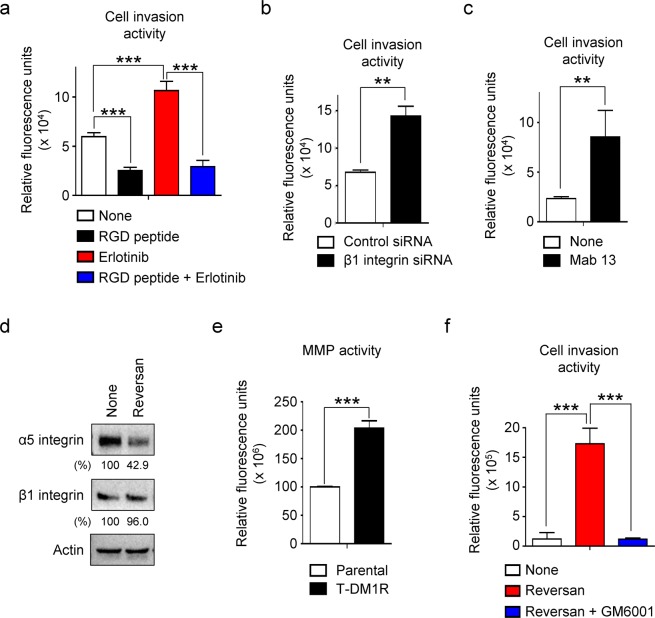
Functional correlation between MRP1 and α5β1 integrin is conserved in breast cancer cells. (**a**) Cell invasion activity of MDA-MB-231 parental cells treated with either none or 5 µM erlotinib for 48 hrs in the media with or without RGD peptide. None vs. RGD peptide: *p*-value, 0.0001; none vs. erlotinib: *p*-value, 0.0007; erlotinib vs. RGD peptide + erlotinib: *p*-value, 0.0001. (**b**) Cell invasion activity of MDA-MB-231 parental cells treated with either control siRNA or β1 integrin siRNA for 48 hrs. Control siRNA vs. β1 integrin siRNA: *p*-value, 0.0003. (**c**) Cell invasion activity of MDA-MB-231 parental cells treated with an inhibitory antibody Mab 13 for 48 hrs. None vs. Mab 13: *p*-value, 0.0079. (**d**) The levels of α5 and β1 integrin expressions were evaluated by Western blot analysis in the WCL of T-DMR-MDA-MB-231 cells treated with either none or 2.5 µM Reversan for 48 hrs. (**e**) Total MMP activities were measured in the WCL of either MDA-MB-231 parental or T-DM1R cells. Parental vs. T-DM1R: *p*-value, <0.0001. (**f**) Cell invasion activity of MDA-MB-231-derived T-DM1R cells treated with either none or Reversan for 48 hrs with or without MMP inhibitor 50 nM GM6001. None vs. Reversan: *p*-value, 0.0003; Reversan vs. Reversan + GM6001: *p*-value, 0.0002.

Since T-DM1R-JIMT1 cells exhibited molecular phenotype that is similar to that in MDA-MB-231 cells, we then used MDA-MB-231 cells as an additional cellular model to further explore functional connections between EGFR and α5β1 integrins and its impact on cell invasion activity. As shown in Fig. 6a, inhibition of EGFR by erlotinib enhanced cell invasion activity in MDA-MB-231 cells, and this enhanced cell invasion activity was rescued by RGD peptide (Fig. 6a). Furthermore, blockage of α5β1 integrin activity by either β1 integrin siRNA or an inhibitory antibody Mab 13 significantly enhanced cell invasion activity in MDA-MB-231 cells (Fig. 6b,c). These observations are reminiscent to the changes in T-DM1R-JIMT1 cells in response to the treatment of erlotinib, RGD peptide, β1 integrin siRNA or Mab 13^10^. The similarity in functional connections between EGFR and α5β1 integrins in both T-DM1R-JIMT1 and MDA-MB-231 cells suggest that carefully targeting both EGFR and integrins may be a novel therapeutic approach to treat both T-DM1R and triple negative breast cancers.

The functional correlation between MRP1 and integrins was also examined in T-DM1R-MDA-MB-231 cells. As shown in Fig. 6d, inhibition of MRP1 activity by reversan caused a dramatical decrease in α5 integrin expression. These results suggest that the functional correlation between MRP1 and α5β1 integrin is conserved, not only in T-DM1R-JIMT1 cells, but also in T-DM1R-MDA-MB-231 cells. Additionally, as consistent with the results in JIMT1-T-DM1R cells, MMP activity was significantly increased in T-DM1R-MDA-MB-231 cells, suggesting that MMP activity is a crucial for enhanced cell invasion activity (Fig. 6e). Figure 6f showed that inhibition of MRP1 activity enhanced cell invasion activity, and this enhanced invasion activity can be reversed by MMP inhibitor, GM6001 (Fig. 6f). Taken together, our findings provide the evidence that upregulation of MRP1 and α5 integrin and downregulation of HER2 are conserved as common mechanisms to contribute to T-DM1 resistance in different breast cancer cell types.

Acquired multiple drug resistance (MDR) is one of major clinical issues in cancer therapy. This study provides useful information for the development of novel targeted therapy for T-DM1-resistant disease that is accompanied by cross-resistance to chemotherapeutic drugs. HER2 and EGFR are very important therapeutic targets for the treatment of cancers. A compensatory mechanism appears to be involved in regulating their expressions in cancer cells treated with either anti-HER2 or anti-EGFR^28,29^. Consistent with our study, it has been recently shown that decrease in HER2 expression causes EGFR upregulation in T-DM1-resistant breast cancer cells^11^. Reversely, inhibition of EGFR activity by the tyrosine kinase inhibitor osimertinib lead an increase in HER2 expression within 24 hrs in non-small cell lung cancer (NSCLC) cells^30^. These studies support the idea that the compensatory mechanism between HER2 and EGFR is conserved among different cancers as a drug resistant mechanism in response to HER family receptor-targeted therapies. Based on data we obtained from T-DM1R-JIMT1 cells, it appears that anti-EGFR agent erlotinib can re-sensitize T-DM1R cells to Dox and combination of erlotinib with reversan can further re-sensitize T-DM1R cells to Dox. Toward this end, it seems that both EGFR and MRP1 could potentially serve as therapeutic targets for the treatment of T-DM-resistant disease that is accompanied with cross-resistance to Dox.

However, effects of erlotinib and reversan on T-DM1R cell growth were different from that on cell invasion activity, such that cell invasion activity is enhanced when EGFR or MRP1 activities is inhibited by erlotinib^10^, reversan, or MRP1 siRNA (Fig. 3b,c). This therapeutic paradox should draw careful attention in clinical studies when a new targeted therapy is tested for the treatment of drug-resistant cancer patients. Different aspects of tumor biology affected by targeted therapeutics, including cancer cell growth/proliferation and cancer cell invasion/metastasis, should be fully characterized. Our data suggest that cancer cell proliferation and invasion are regulated by different mechanisms and that inhibition of cancer cell proliferation is not always positively co-related with blocking cancer metastasis, rather promoting it. Therefore, novel drugs that block cancer cell metastasis is urgently needed to overcome drug-resistant disease.

## Materials and Methods

### Cells and therapeutic drugs

JIMT1-derived T-DM1R cells were established and maintained as described previously^10^. MDA-MB-231, BT-474 and BT-549 cells were cultured in RPMI-1640 containing 10% FBS. Procedures and time lines for establishing BT-474, MDA-MB-231 and BT-549-derived T-DM1 resistant cells were described in Figs 4b and 5a, respectively. All T-DM1R cell lines were maintained in the presence of T-DM1. T-DM1, doxorubicin and erlotinib were purchased as described previously^10^. Reversan was purchased from TOCRIS (cat#3722). GM6001 was purchased from EMD Millipore (cat#CC1010).

### Immunofluorescent staining

The procedures for immunofluorescent staining and information regarding α5 and β1 integrin antibodies were described previously^10^. Immunofluorescent staining images were quantified using ImageJ software (NIH).

### siRNA transfection

The detailed procedures of siRNA transfection were described previously^10^. siRNAs against human β1 integrin (cat#L-004506–00–0005), human MRP1 (cat#L-007308-00-0005), and non-targeting control siRNA (cat#D-001810-10-20) were purchased from GE Dharmacon.

### Cell invasion assay

The detailed procedures were described previously^10^.

### Cell adhesion/spreading assay

The detailed procedure for cell adhesion/spreading assay was described previously^31^. Briefly, 6-well plates were coated with fibronectin (10 µl/ml, Sigma) in PBS at 4 °C overnight. After washing with PBS, BT-474 parental and T-DM1R-BT-474 cells were suspended using trypsin-EDTA and allowed to recover from the enzymatic treatment by incubation for 30 min 10% FBS / RPMI-1640 at 37 °C. Then, 0.5 × 10^5^ cells were seeded in the fibronectin-coated wells. After incubation for 4 hrs at 37 °C, adherent cells were counted in randomly selected regions (n = 5 to 7). The images were taken by a CKX41 microscope (Olympus) with an INFINITY1 CMOS digital camera (Teledyne Lumenera).

### MMP activity

MMP Activity Assay Kit (Abcam, cat#112146) was used for measuring total MMP activities in supernatant of cell lysate. The detailed procedures were followed by the manufacturer’s protocol. Briefly, 1 × 10^6^ cells were seed in a well of 6-well plate and cultured overnight. After rinse with ice-cold TBS once, an ice-cold assay buffer provided in the kit was added to cells, and then they were immediately collected by a cell scraper. After centrifugation to remove insoluble cellular debris, the whole cell supernatants (WCS) were collected. Protein concentration in WCS was measured by O.D. 280 nm, and protein concentration of samples was adjusted to equal. After mixing the WCS with MMP Green Substrate provided in the kit, the relative fluorescence units (RFU) were measured by using a fluorescence plate reader (VICTOR X3, PerkinElmer).

### Statistical analysis

GraphPad Prism was used for statistical analysis. Student’s *t*-test was employed to determine statistical significance (*p-value < 0.05; **p-value < 0.01; ***p-value < 0.001). Data are expressed as mean ± SD.

## Supplementary information

Supplemental information
