# Supplementary material for: Cell proliferation and invasion are regulated differently by EGFR and MRP1 in T-DM1-resistant breast cancer cells
Source: Sci Rep. 2019 Nov 8;9:16383. doi: 10.1038/s41598-019-52797-z (PMC6842003; doi:10.1038/s41598-019-52797-z)

Cell proliferation and invasion are regulated differently by EGFR and MRP1 in T-DM1-resistant breast cancer cells

Yukinori Endo[1], Sarah Lyon[1], Yi Shen[1], Nishant Mohan[1], Wen Jin Wu[1,*]

Affiliation:

[1]Division of Biotechnology Review and Research I, Office of Biotechnology Products, Office of Pharmaceutical Quality, Center for Drug Evaluation and Research, U.S. Food and Drug Administration (FDA), Silver Spring, MD 20993, USA

*Corresponding author: Wen Jin Wu, Building 52/72, Room 2310, 10903 New Hampshire Avenue, Silver Spring, MD, 20993 USA. Email: Wen.Wu@fda.hhs.gov; Phone: 240-402-6715

For Fig. 1c

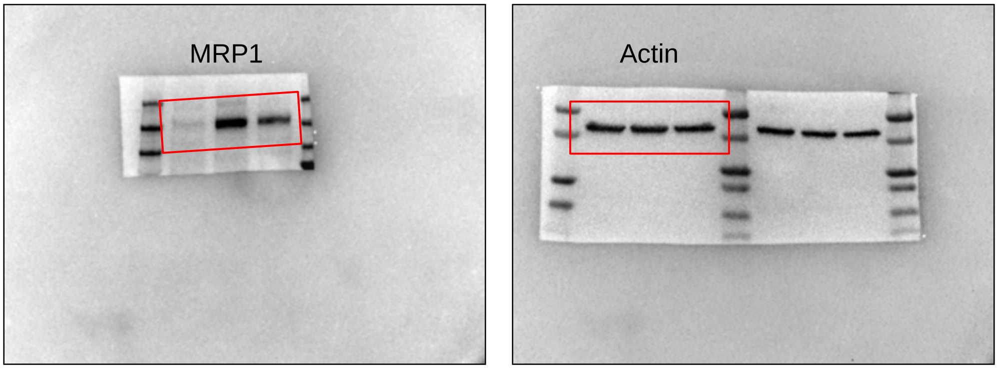

For Fig. 1d

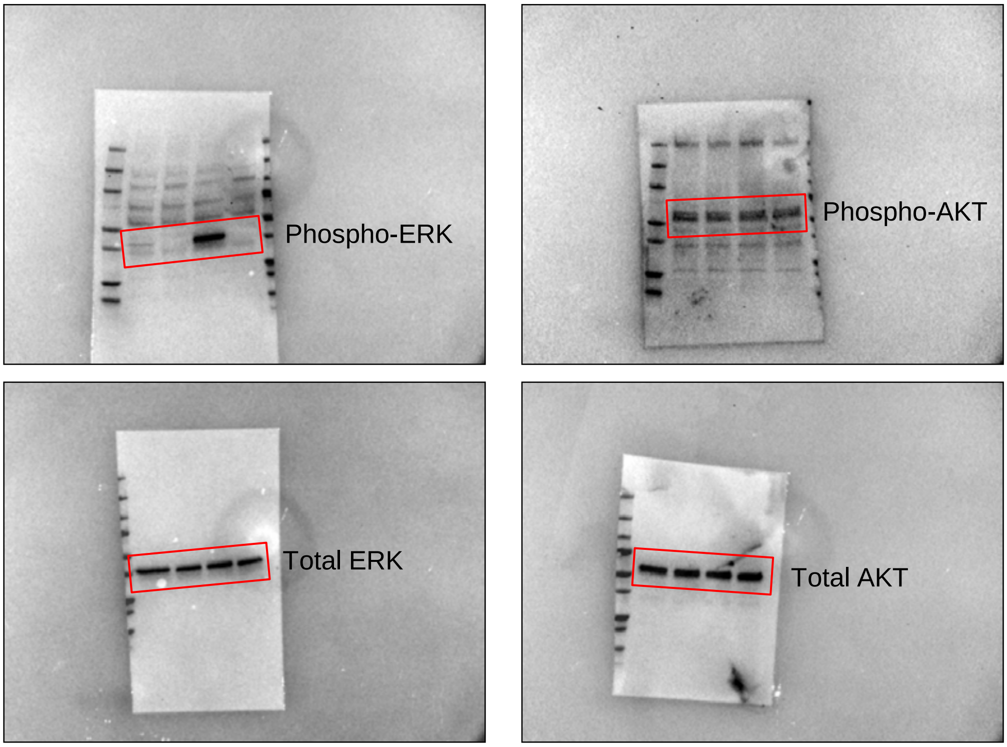

For Fig.1e

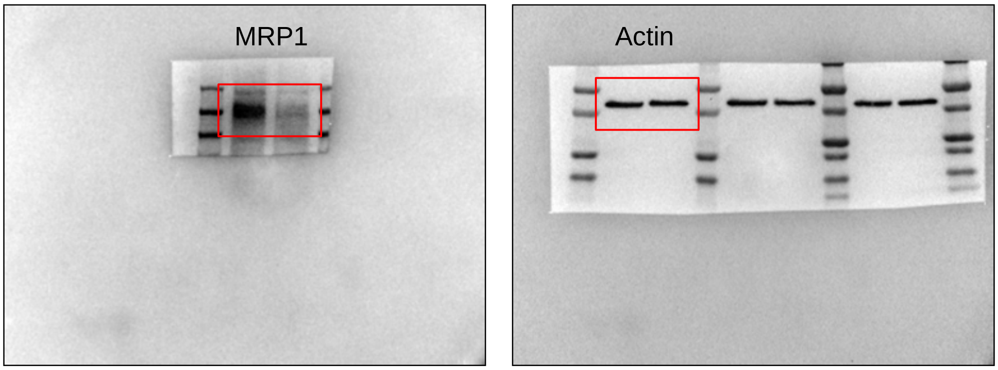

Supplementary information 1

For Fig. 2b

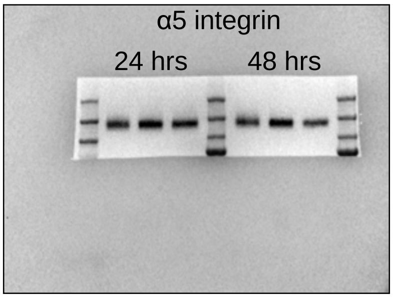

with protein marker

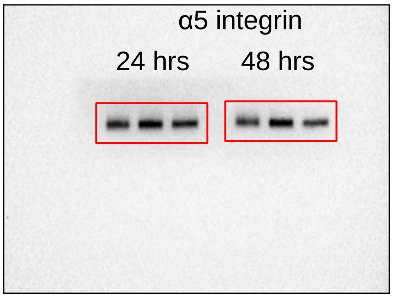

without protein marker

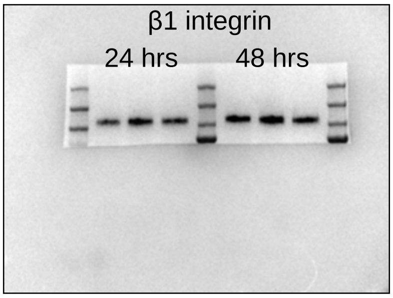

with protein marker

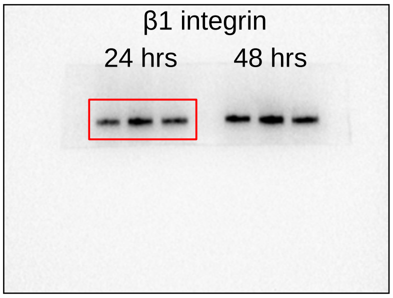

without protein marker

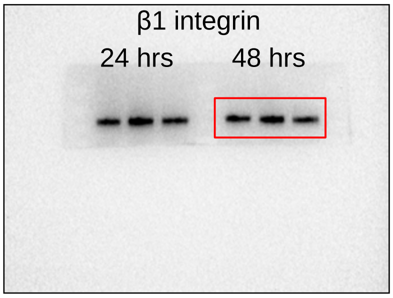

without protein marker

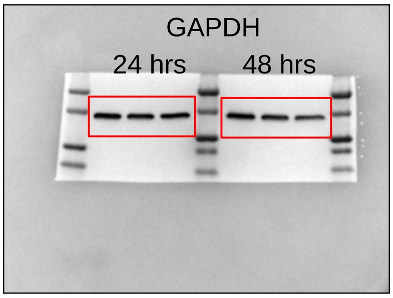

For Fig. 2f, g

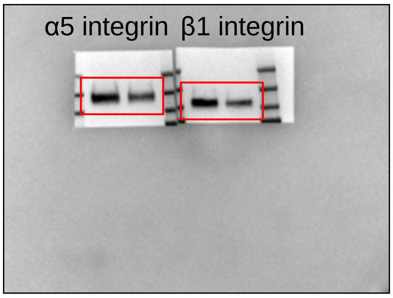

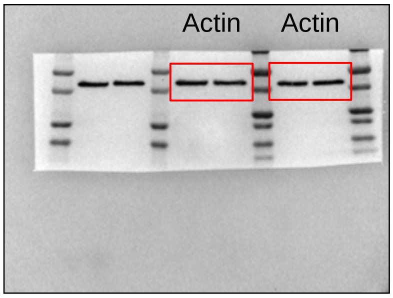

Supplementary information 2

For Fig. 3a

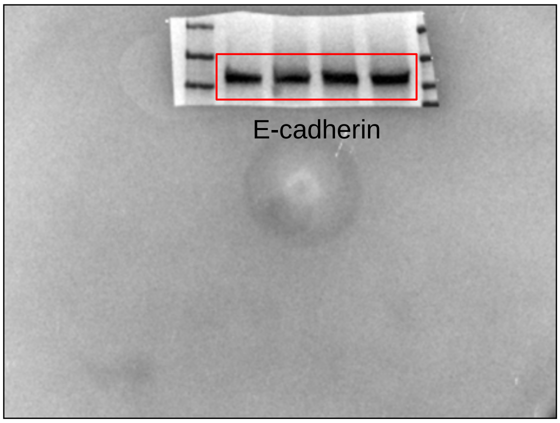

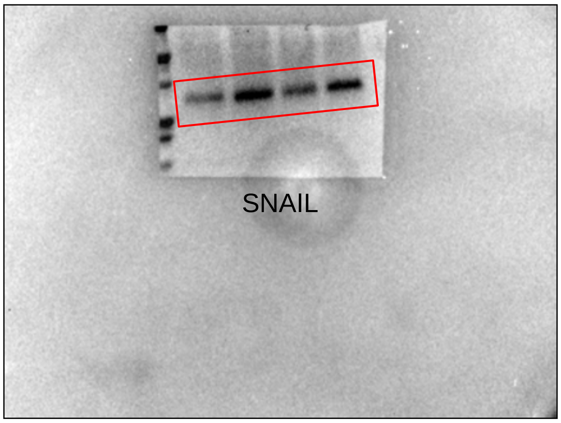

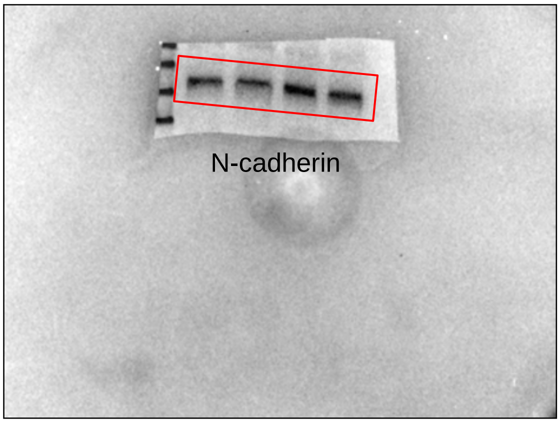

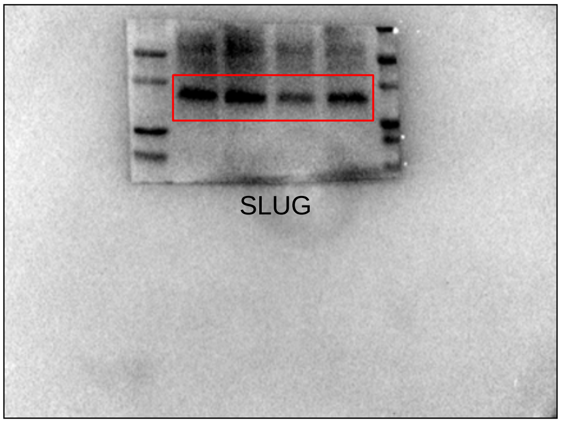

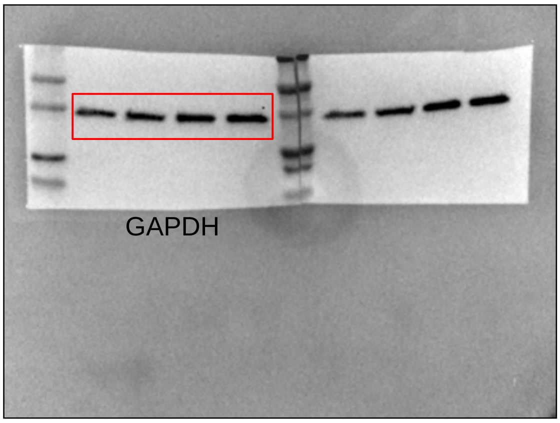

Supplementary information 3

For Fig. 4a

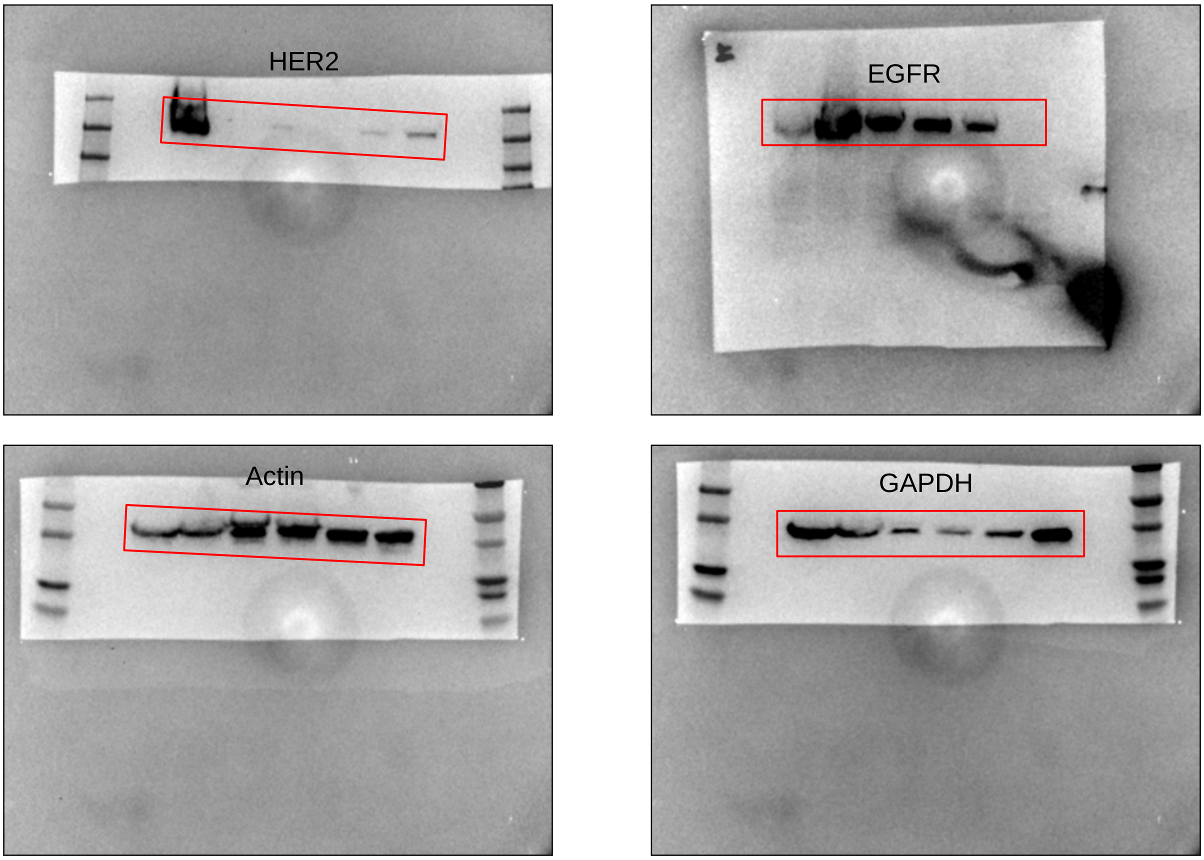

For Fig. 4e

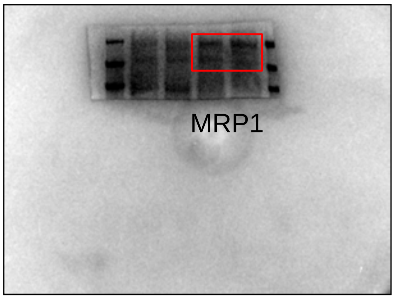

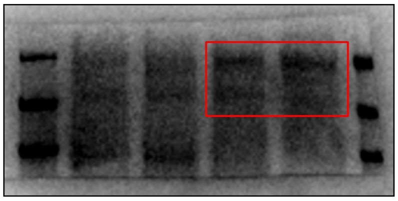

MRP1
(Magnified)

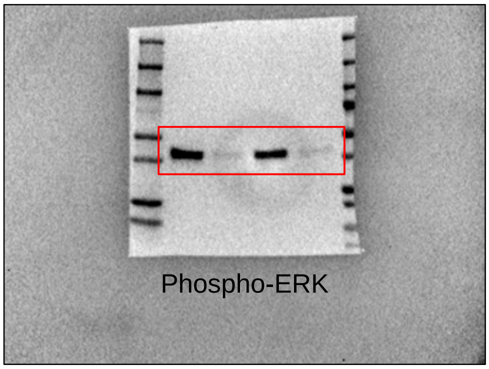

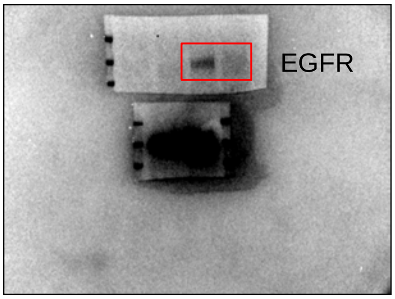

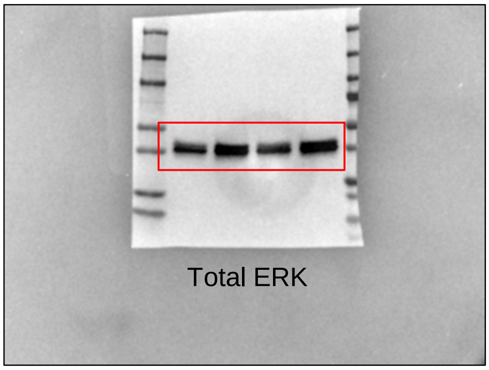

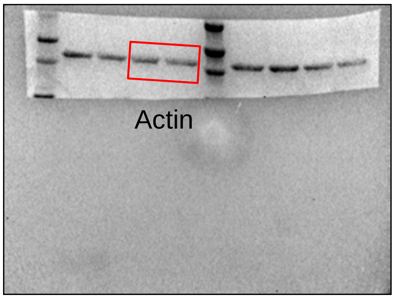

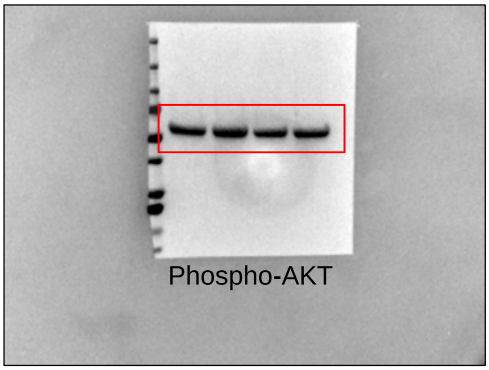

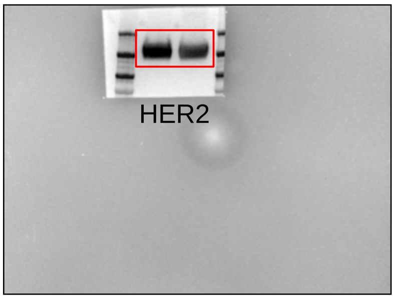

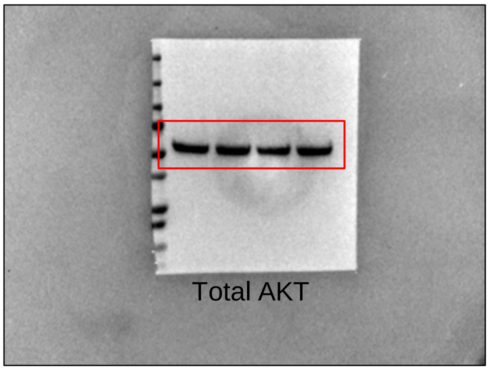

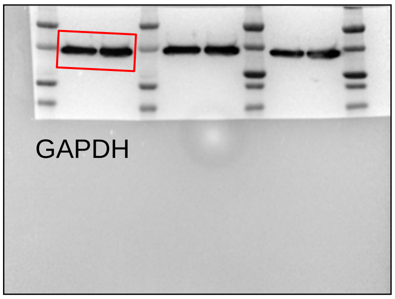

For Fig. 5e

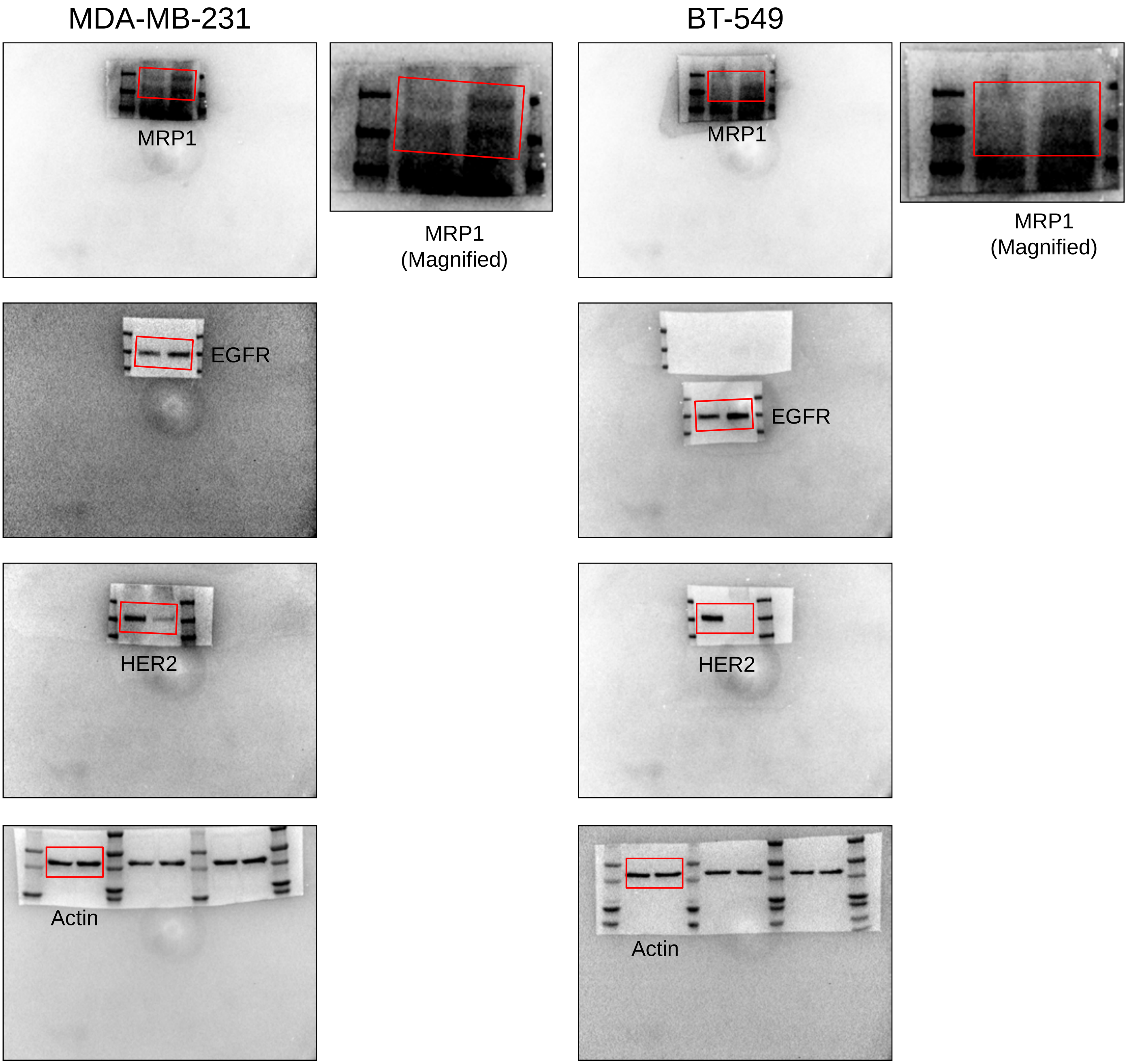
MDA-MB-231
BT-549
MRP1
MRP1
(Magnified)
MRP1
MRP1
(Magnified)
EGFR
EGFR
HER2
HER2
Actin
Actin

For Fig. 5f

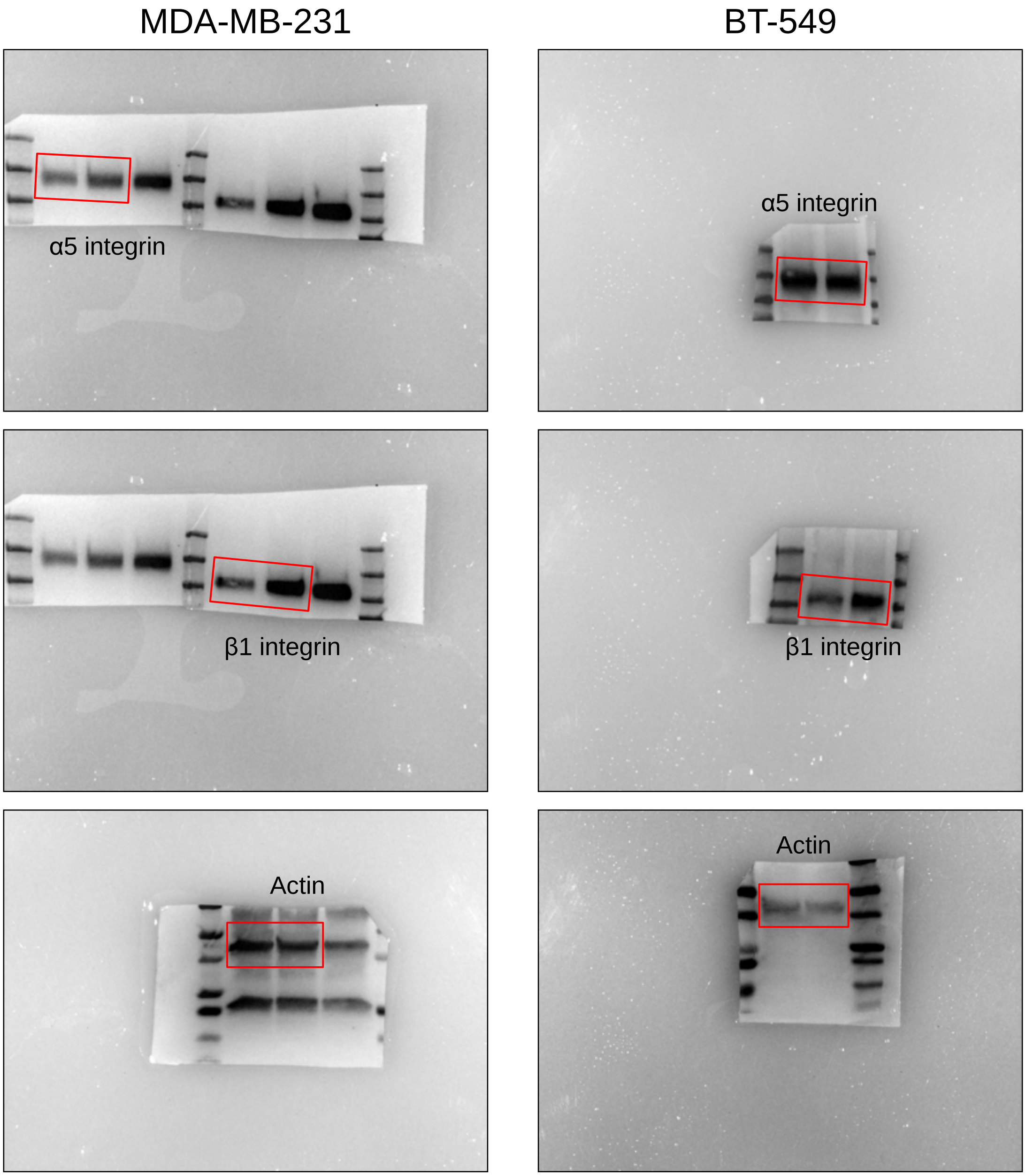
MDA-MB-231
BT-549
α5 integrin
α5 integrin
β1 integrin
β1 integrin
Actin
Actin

For Fig. 6d

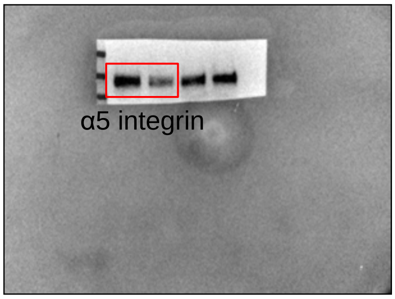

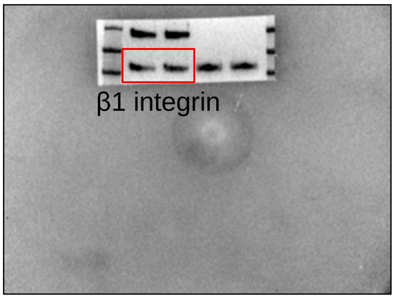

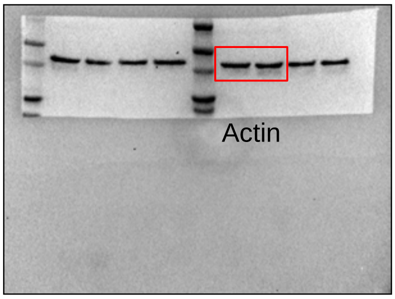

Supplement: Supplementary file 1 — Supplemental information [file 41598_2019_52797_MOESM1_ESM.pdf]
